# The c-MYC Protooncogene Expression in Cholesteatoma

**DOI:** 10.1155/2014/639896

**Published:** 2014-02-10

**Authors:** Enikő Palkó, Szilárd Póliska, Zsuzsanna Csákányi, Gábor Katona, Tamás Karosi, Frigyes Helfferich, András Penyige, István Sziklai

**Affiliations:** ^1^Department of Otorhinolaryngology Head and Neck Surgery, B-A-Z County Hospital and University Hospital, Szentpéteri Kapu 72-76, Miskolc 3526, Hungary; ^2^Department of Biochemistry and Molecular Biology Research Center for Molecular Medicine, University of Debrecen, Medical and Health Science Center, Nagyerdei Körút 98, Debrecen 4032, Hungary; ^3^Department of Otorhinolaryngology, Children's Hospital Heim Pál, Üllői Ut 86, Budapest 1083, Hungary; ^4^Department of Otorhinolaryngology Head and Neck Surgery, Military Hospital, Podmaniczky Utca 109-111, Budapest 1062, Hungary; ^5^Department of Human Genetics, University of Debrecen, Medical and Health Science Center, Nagyerdei Körút 98, Debrecen 4032, Hungary; ^6^Department of Otorhinolaryngology, Head and Neck Surgery, University of Debrecen, Medical and Health Science Center, Nagyerdei Körút 98, Debrecen 4032, Hungary

## Abstract

Cholesteatoma is an epidermoid cyst, which is most frequently found in the middle ear. The matrix of cholesteatoma is histologically similar to the matrix of the epidermoid cyst of the skin (atheroma); their epithelium is characterized by hyperproliferation. The c-MYC protooncogene located on chromosome 8q24 encodes a transcription factor involved in the regulation of cell proliferation and differentiation. Previous studies have found aneuploidy of chromosome 8, copy number variation of c-MYC gene, and the presence of elevated level c-MYC protein in cholesteatoma. In this study we have compared the expression of c-MYC gene in samples taken from the matrix of 26 acquired cholesteatomas (15 children and 11 adults), 15 epidermoid cysts of the skin (atheromas; head and neck region) and 5 normal skin samples (retroauricular region) using RT-qPCR, providing the first precise measurement of the expression of c-MYC gene in cholesteatoma. We have found significantly elevated c-MYC gene expression in cholesteatoma compared to atheroma and to normal skin samples. There was no significant difference, however, in c-MYC gene expression between cholesteatoma samples of children and adults. The significant difference in c-MYC gene expression level in cholesteatoma compared to that of atheroma implies a more prominent hyperproliferative phenotype which may explain the clinical behavior typical of cholesteatoma.

## 1. Introduction

Several types of keratinous cysts are known, such as epidermal cyst of the skin (atheroma) and cholesteatoma. Cholesteatoma is an epidermoid cyst, characterized by hyperproliferation of multilayered keratinizing squamous epithelium, causing bone erosion and destruction in the middle ear. Atheroma is a benign tumor. The epithelium wall of these cysts is called matrix; the surrounding tissue, the perimatrix or lamina propria, is the peripheral part of the cyst. [1–3]. Although epidermoid cyst of the skin and cholesteatoma are histologically very similar, the clinical behavior of cholesteatoma differs markedly from that of atheroma [4].

Several theories can be found in the literature to explain the development and aggressive behavior of cholesteatoma, among them changes in the physiological and anatomic conditions of the middle ear or molecular abnormalities [1, 2, 5–8].

According to recent studies, the cholesteatoma has an assumed neoplastic behavior characterized by chromosomal abnormalities [9]. Previous studies had shown aneuploidy of chromosome 8 and as a consequence copy number variation of c-MYC gene and the presence of elevated level c-MYC protein in cholesteatoma [9–12]. The c-MYC gene is a protooncogene, located on chromosome 8q24. It is well known that deregulated expression of this gene plays a significant role in tumorogenesis; genomic alterations affecting chromosome 8q24 are frequent in cancers [13, 14].

These reports, however, did not provide an exact measurement of the expression of the c-MYC gene itself. The characteristic alterations present in cholesteatoma indicate abnormal c-MYC gene expression and could warrant for the precise determination of the c-MYC gene expression level in cholesteatoma samples.

Compared to the methods used in previous studies RT-qPCR provides a more accurate measurement of gene expression level. It enables to detect and quantify the extent of abnormal c-MYC gene expression that could be present in cholesteatoma.

The aim of our study was to determine the expression level of the c-MYC protooncogene, a plausible causative factor of the hyperproliferation of the epithelium (matrix) in cholesteatoma. According to our knowledge, this is the first study that uses RT-qPCR methodology to measure c-MYC gene expression in cholesteatoma samples and the first to compare c-MYC gene expression in an inflammatory destructive cholesteatoma with that of the histologically similar benign epidermoid cyst of the skin, atheroma. Normal skin samples were used as controls in our experiments.

## 2. Materials and Methods

### 2.1. Patients and Tissue Sample Preparation

All samples were obtained with informed patient consent and with approval from the Research Ethics Committee of University of Debrecen Medical and Health Science Center that approved the clinical protocol and the study (protocol number 3047-2009).

Our study populations consisted of 26 patients with acquired cholesteatoma (11 females and 15 males), 15 patients with atheroma (head-neck region), and 5 normal skin samples (retroauricular region). The eardrums were perforated in all cholesteatoma patients, and all patients underwent primary or secondary surgery. The age of the cholesteatoma patients ranged between 4 and 65 years (average: 23.4 years). Patients were divided into a pediatric (15 cases; 0–18 years) and an adult group (11 cases; over 19 years). The clinical samples were provided by the Children's Hospital Heim Pál, Regional Educational Hospital “Jósa András,” and the University of Debrecen Medical and Health Science Center. The diagnosis of cholesteatoma and atheroma in all specimens was confirmed by histopathologic examination.

All samples were surgically removed. Immediately following surgery, the samples were soaked and fixed in RNA*later* RNA Stabilization Reagent (Life Technologies) and stored at 4°C until RNA extraction.

### 2.2. RNA Extraction

Before RNA extraction the excess RNA*later* solution was blotted off from the samples and the matrixes of cholesteatoma and the atheroma specimens were carefully and manually cleaned from the surrounding tissues. Optimally 70 mg tissue sample was homogenized in TRI Reagent with a rotor-stator tissue homogenizer and total RNA was extracted from the specimens using the RiboPure kit following the manufacturer's instructions (Ambion (Europe) LTD, Huntingdon, UK). The concentration and quality of the RNA samples were assessed by a NanoDropTM 1000A spectrophotometer (Thermo Scientific, Wilmington, USA).

### 2.3. cDNA Synthesis and Real-Time PCR

First-strand synthesis for real-time PCR cDNA preparation was performed on 2 *μ*g of total RNA, using the High Capacity cDNA Reverse Transcription Kit with RNase inhibitor (Applied Biosystems) in a final volume of 20 *μ*L. The samples were incubated at 25°C for 10 min and 37°C for 120 min; the reverse transcriptase was inactivated at 85°C for 5 min and cooled at 0°C for an additional 5 min.

Determination of the target c-MYC gene mRNA expression was performed on an ABI Prism 7900HT Sequence Detection System (Applied Biosystems) by fluorescent TaqMan methodology using prevalidated TaqMan Gene Expression Assays (*MYC *Assay ID Hs00153408_m1). Gene expression quantitation was performed according to the manufacturer's instructions; briefly a total of 20 *μ*L of PCR mix containing TaqMan Universal PCR master mix with AmpliTaq Gold DNA Polymerase, TaqMan Gene Expression Assay mix, and 4 ng of cDNA was used. Amplification was performed for 40 cycles, including denaturation at 95°C for 15 seconds and annealing at 60°C and extension at 72°C for 60 and 30 seconds, respectively. Relative gene expression levels were calculated by the comparative critical threshold method (2^−ΔΔCT^) using PPIA as a housekeeping gene to normalize expression levels. The housekeeping gene PPIA was coamplified with c-MYC on the same plate. Results are expressed as relative increase or decrease compared to the normal control group.

### 2.4. Statistical Analysis

The nonparametric Mann-Whitney *U* test was performed to assess the statistical significance of differences between relative mRNA levels. The GraphPad Prism software was used for the analysis and statistical significance was defined as *P* < 0.05.

## 3. Results

In order to compare c-MYC gene expression 26 acquired cholesteatoma samples (15 children and 11 adults), 15 atheroma samples, and 5 normal skin samples were collected. The demographic data and surgical parameters of cholesteatoma patients are summarized in Table 1. Typically cholesteatoma extended to the atticus and antrum; in 24 out of the 26 patients the ossicular chain was destructed and in 2 cases it was intact. The recurrent rate of cholesteatoma was higher in the pediatric group compared to that of the adult group (31% and 27%, resp.).

**Figure 1:**
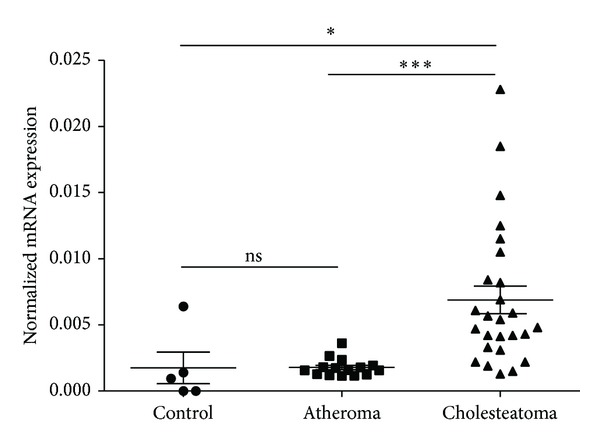
c-MYC gene expression in control, atheroma, and cholesteatoma samples. The housekeeping gene cyclophilin A (PPIA) was used to normalize mRNA expression levels. The mean value and the standard deviation of the data are shown on the figure. The significance levels are *P = 0.0125 and ***P = 0.0001; ns: not significant.

To quantitate c-MYC gene expression, total RNA content was extracted from the homogenized surgical samples and first-strand cDNA was generated from equal amounts of RNA. C-MYC target sequence was amplified and quantitated by a TaqMan methodology based real-time PCR (RT-PCR) assay. Our RT-QPCR data show that the PPIA normalized c-MYC expression was identical in the control and atheroma samples (*μ* ± SD = 1.17 × 10^−3^ ± 1.01 × 10^−3^) in normal skin and 1.8 × 10^−3^ ± 3.2 × 10^−5^ in atheroma (*P* = 0.137). In cholesteatoma samples, however, c-MYC expression level was significantly elevated (*μ* ± SD = 6.9 × 10^−3^ ± 1.08 × 10^−3^) compared to that in atheroma (*P* = 0.0001) and in control samples (*P* = 0.012) (Figure 1).

**Figure 2:**
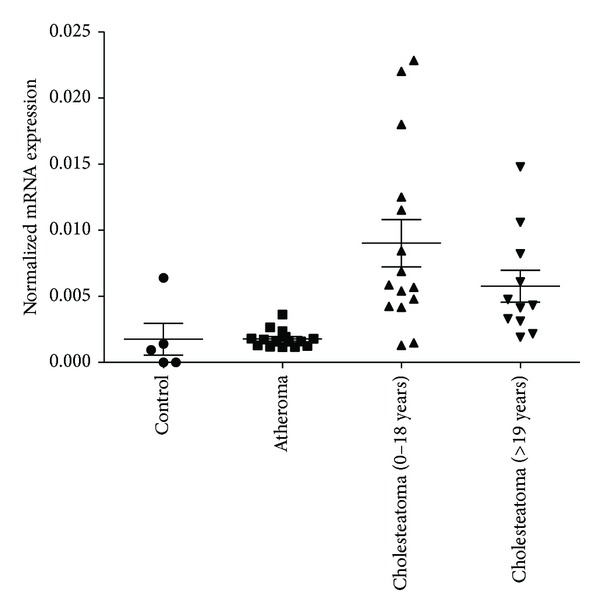
The distribution of c-MYC gene expressions values in samples of children and adult cholesteatoma patients compared to that of atheroma and control samples. The housekeeping gene cyclophilin A (PPIA) was used to normalize mRNA expression levels. Mean values and standard deviations are shown on the figure. The significance levels of the data are given in the text.

**Figure 3:**
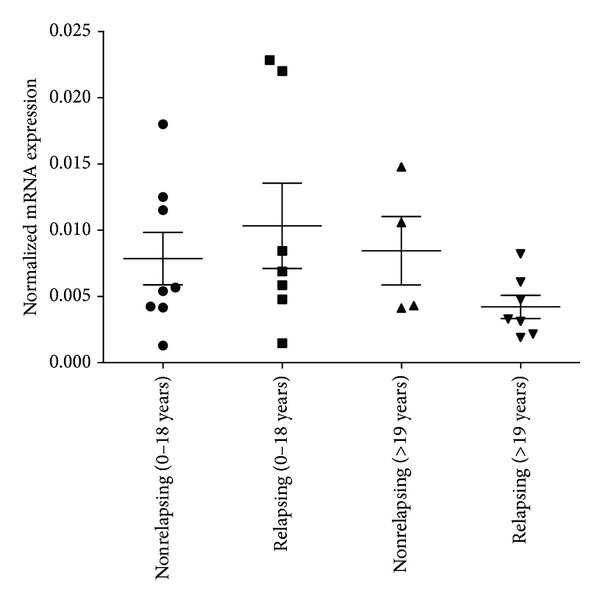
The c-MYC gene expression values in samples of relapsing and nonrelapsing cholesteatomas in children and in adult patients. There was no significant difference in the expression of the c-MYC gene between these groups. The housekeeping gene cyclophilin A (PPIA) was used to normalize mRNA expression levels. Mean values and standard deviations are shown on the figure.

Cholesteatoma patients were divided into two groups: a pediatric (age < 18) and an adult group (age > 19). Mean relative expression level of c-MYC in cholesteatoma samples of children was slightly higher than in the adults (*μ* ± SD = 9.1 × 10^−3^ ± 1.17 × 10^−3^ and 6.3 × 10^−3^ ± 1.0 × 10^−3^, resp.); however the data showed wider distribution in the pediatric samples. The difference in c-MYC expression was not significant between the two cholesteatoma groups (*P* = 0.195). c-MYC expression was significantly higher in both cholesteatoma groups compared to that of atheroma specimens and to controls (cholesteatoma-atheroma comparison: *P*_children_ = 0.001 and *P*_adults_ = 0.002; cholesteatoma-control comparison: *P*_children_ = 0.0145 and *P*_adults_ = 0.0312) (Figure 2). The highest c-MYC expression was found in children with recurrent cholesteatoma; however, the difference was not significant in any of the pairwise comparisons (Figure 3).

## 4. Discussion

Several types of keratinous cysts are known, which are characterized by different keratinisation mechanisms. The matrix of the keratinous cyst contains mainly epithelial cells (keratinous cells) with different differentiation states [4]. Cholesteatoma is a multilayered keratinizing squamous cyst; it is characterized by uncoordinated cell proliferation, invasion of surrounding tissues, altered differentiation states, intense inflammation, and recurrence, causing several clinical complications. Atheroma is a keratinous cyst too. It is a benign tumor; it can be manifested anywhere in the body without serious complications [1–3]. Atheroma is delineated by a multilayered epidermal-like epithelium including a characteristical granular layer, and the content of the cyst is laminated keratin. The matrixes of the cholesteatoma and atheroma are histologically very similar; however, their surrounding environment and behavior are different [1–4]. The cholesteatomatous suppurative otitis media is a chronic inflammatory process and numerous studies described the involvement of different inflammatory mediators (EGF, TGF-alpha, beta, IL-6, IL-1, and GM-CSF), cell surface markers, and adhesion molecules (1,6 integrin, ICAM ELAM) in the pathomechanism of the disease. The interactions among these mediators could initiate modified signal transduction pathways in keratinocytes of cholesteatoma leading to a higher proliferation rate and a modified cell differentiation pathway [2, 9, 15–19]. However, the exact etiopathogenesis of the cholesteatoma is still not known.

Several recent studies have found chromosome aberrations in cholesteatoma, and this phenomenon might suggest the malfunction of factors involved in the regulation of cell cycle, like tumor suppressor genes or protooncogenes [9–12, 19]. Ozturk and coworkers showed the aneuploidy of chromosome 8 and subsequent copy number variation of the c-MYC gene in cholesteatoma [11]. Increased level of c-MYC protein was also detected by immunohistochemistry in cholesteatoma [12].

The c-MYC gene is located on chromosome 8q24; it codes for a transcription factor that plays an important role in cell cycle progression, in the regulation of cell proliferation, differentiation, and apoptosis. Deregulated expression of this gene has been associated with a variety of tumors, like lymphomas, kidney, lung, colon tumors, and melanomas. C-MYC protooncogene is frequently dysfunctional in head and neck cancer cells and it might be involved in hyperproliferation of epithelial cells [13, 14]. Since abnormal expression of c-MYC occurs in several tumors, its expression level has prognostic importance.

The c-MYC protein is a multifunctional transcription factor involved in the activation of DNA replication; overexpression of this protooncogene may induce chromosome aberrations and an overall dysfunction in the regulation of the cell cycle [13, 14, 20, 21].

In our study we examined the c-MYC expression in the matrix of two types of keratinous cysts. On the basis of clinical data, the 26 acquired cholesteatomas were divided into two groups, a pediatric and an adult group. Both groups were subdivided into patients with relapsing and nonrelapsing group. We have found higher recurrence rate in the pediatric group compared to that of adults.

In the present study we have provided a quantitative assessment of c-MYC gene expression for epidermal cysts using RT-QPCR methodology. Our results showed a significantly elevated expression of c-MYC gene in cholesteatoma, compared to that of atheroma and normal skin samples.The expression level of c-MYC gene in pediatric cholesteatoma was higher than in adults. The highest c-MYC expression level was found in children with recurrent cholesteatoma. According to our opinion the c-MYC expression level is a prognostic factor in cholesteatoma, indicating the clinical aggressiveness and the susceptibility of relapse.

Welkoborsky et al. examined 40 adults and 14 children who acquired cholesteatomas. Immunohistochemical methods were used to determine the expression of proliferation and cell surface markers (MIB-1 and PCNA markers). The cell proliferation indices were higher in both groups compared to normal auditory canal skin samples; however, the highest values were detected for the pediatric group than for the adult one. On the basis of these results, the pediatric cholesteatomas can be characterized with more aggressive proliferative phenotype [18]. This examination is in agreement with the increased c-MYC expression in childhood cholesteatomas found in our study. This genetic deviation was also supported by the clinical data of our study population.

Several research groups have found an elevated level of certain inflammatory mediators involved in the inflammation processes in cholesteatoma [15–17]. On the basis of patients' data Welkoborsky et al. concluded that the intensity of inflammation process was higher in pediatric cholesteatoma than in adults [18].

Based on these results, one might conclude that the increased expression of the c-MYC in cholesteatoma is induced by inflammation. However, according to the literature, inflammation factors do not play a direct regulatory role in the expression of the c-MYC gene. Interestingly recent results show that increased c-MYC expression can cause mitochondrial dysfunction leading to reactive oxygen species production and as a consequence can fuel inflammation [22]. According to the “field cancerization” theory described by Slaughter and cited by Schwartz et al. tumorogenesis is a multiple stage process [23]. Chronic inflammation can increase cancer risk (extrinsic pathway); however, genetic alterations (intrinsic pathway and oncogene activation) are also required for tumorogenesis [24]. A previous case study has reported that after the removal of cholesteatoma a squamous epithelium carcinoma developed in the middle ear of the patient [25].

The elevated c-MYC level reported in this study might have a dual role; it promotes cell proliferation and can sustain the chronic inflammation process in cholesteatoma.

## 5. Conclusion

The increased c-MYC expression level in the cholesteatoma matrix compared to that of the atheroma matrix might explain the more aggressive behavior of cholesteatoma that results in clinical complications and it also explains its more intensive proliferation. The elevated c-MYC expression could lead to increased cell proliferation rate which is associated with frequent relapse. Our finding that c-MYC expression was higher in the cholesteatoma matrix of children could explain the higher recurrence rate in this group compared to that of the adult group. Based on the elevated c-MYC expression level, cholesteatoma is similar to a neoplastic malformation; however, further experiments are needed to clarify its role in the pathogenesis of cholesteatoma.

## Figures and Tables

**Table 1:** Demography of cholesteatoma patients and surgical findings.

Sample number	Age (year)	Gender*	Expansion	Chain of auditory ossicles	Recurrence
1	25	M	Atticus + antrum	Destroyed	Yes
2	11	F	Atticus + mastoid	Destroyed	No
3	32	F	Atticus + antrum	Destroyed	No
4	42	M	Atticus	Destroyed	No
5	58	F	Atticus + antrum	Destroyed	No
6	14	M	Atticus + mastoid	Destroyed	Yes
7	8	M	Atticus	Destroyed	Yes
8	11	M	Atticus, mastoid	Intact	Yes
9	22	F	Mastoid	Destroyed	Yes
10	60	F	Atticus	Destroyed	Yes
11	62	M	Atticus, mastoid	Destroyed	No
12	12	M	Atticus, mastoid	Destroyed	No
13	8	M	Atticus, antrum	Destroyed	No
14	16	F	Atticus, antrum	Intact	No
15	34	F	Atticus, mastoid	Destroyed	Yes
16	11	M	Atticus, mastoid	Destroyed	Yes
17	16	M	Attius, mastoid	Destroyed	Yes
18	10	F	Atticus, mastoid	Destroyed	Yes
19	27	F	Atticus, mastoid	Destroyed	Yes
20	31	M	Atticus, mastoid	Destroyed	Yes
21	8	F	Atticus, mastoid	Destroyed	No
22	4	M	Atticus, antrum	Destroyed	Yes
23	6	M	Atticus	Destroyed	No
24	4	M	Atticus	Destroyed	No
25	11	F	Atticus	Destroyed	Yes
26	65	M	Atticus, mastoid	Destroyed	Yes

*F: female and M: male.
